# Supercritical CO_2_ Synthesis of Freestanding Se_1-*x*_S_*x*_ Foamy Cathodes for High-Performance Li-Se_1-*x*_S_*x*_ Battery

**DOI:** 10.3389/fchem.2021.738977

**Published:** 2021-07-28

**Authors:** Chengwei Lu, Ruyi Fang, Kun Wang, Zhen Xiao, G. Gnana kumar, Yongping Gan, Xinping He, Hui Huang, Wenkui Zhang, Yang Xia

**Affiliations:** ^1^ College of Materials Science and Engineering, Zhejiang University of Technology, Hangzhou, China; ^2^ Institute of Optoelectronic Materials and Devices, China Jiliang University, Hangzhou, China; ^3^ Department of Physical Chemistry, School of Chemistry, Madurai Kamaraj University, Madurai, India

**Keywords:** Se1-xSx, N-doped carbon foam, supercritical CO2, high areal capacity, Li-Se1-xSx batteries

## Abstract

Selenium-sulfur solid solutions (Se_1-*x*_S_*x*_) are considered to be a new class of promising cathodic materials for high-performance rechargeable lithium batteries owing to their superior electric conductivity than S and higher theoretical specific capacity than Se. In this work, high-performance Li-Se_1-*x*_S_*x*_ batteries employed freestanding cathodes by encapsulating Se_1-*x*_S_*x*_ in a N-doped carbon framework with three-dimensional (3D) interconnected porous structure (NC@SWCNTs) are proposed. Se_1-*x*_S_*x*_ is uniformly dispersed in 3D porous carbon matrix with the assistance of supercritical CO_2_ (SC-CO_2_) technique. Impressively, NC@SWCNTs host not only provides spatial confinement for Se_1-*x*_S_*x*_ and efficient physical/chemical adsorption of intermediates, but also offers a highly conductive framework to facilitate ion/electron transport. More importantly, the Se/S ratio of Se_1-*x*_S_*x*_ plays an important role on the electrochemical performance of Li- Se_1-*x*_S_*x*_ batteries. Benefiting from the rationally designed structure and chemical composition, NC@SWCNTs@Se_0.2_S_0.8_ cathode exhibits excellent cyclic stability (632 mA h g−1 at 200 cycle at 0.2 A g^−1^) and superior rate capability (415 mA h g^−1^ at 2.0 A g^−1^) in carbonate-based electrolyte. This novel NC@SWCNTs@Se_0.2_S_0.8_ cathode not only introduces a new strategy to design high-performance cathodes, but also provides a new approach to fabricate freestanding cathodes towards practical applications of high-energy-density rechargeable batteries.

## Introduction

Lithium-sulfur (Li-S) batteries are considered as promising next-generation electrochemical energy-storage systems in view of their high theoretical energy density (2600 W h kg^−1^), environmental friendliness and natural richness of sulfur (Yao et al., 2017; Zheng et al., 2020; Yuan et al., 2021a; Yuan et al., 2021b; Sun et al., 2021). Although great progress has been made, the widespread practical application of Li-S battery is still facing issues of the insulation property of natural sulfur (5 × 10^−30^ S m^−1^, 25°C), the serious volume effect in the cycle process, and the dissolution of the intermediate polysulfide, leading to the low sulfur utilization, fast capacity decay and poor cycle stability (Zhang et al., 2020).

As a congener of element S, Se has similar chemical properties with S, such as high theoretical volumetric capacity (3,253 mA h cm^−3^, *ρ* = 4.81 g cm^−3^), which is suitable for mobile devices and hybrid electric vehicles with strict restrictions on battery volume (Lin et al., 2021; Sun et al., 2021). Meanwhile, selenium is a semiconductor with much higher electronic conductivity (1 × 10^−3^ S m^−1^) than sulfur, which is conducive to excellent kinetic behavior (Zhang et al., 2020). Nevertheless, in the current research stage of Li-Se batteries, there are still many problems in Se cathode materials, such as relatively unfavorable higher cost and lower gravimetric capacity (675 mA h g^−1^) when compared to Li-S batteries (1,675 mA h g^−1^) (Fang et al., 2018b). In order to offset the drawbacks of Se and S and complement each other’s advantages, a solid solution of Se and S (Se_1-*x*_S_*x*_, 0 < *x* < 1) has been proposed as high-performance cathode materials for lithium storage. Se_1-*x*_S_*x*_ is a class of chemical compounds with different Se-S ratios, which not only owns a higher theoretical capacity than pure Se, but also has increased electronic conductivity and accelerated reaction kinetics than pristine S (Abouimrane et al., 2012; Wei et al., 2016; Xu et al., 2019).

However, similar to S, Se_1-*x*_S_*x*_ cathode materials also suffer from poor cycle lifespan and low Coulombic efficiency due to the dissolution and shuttling of intermediates (Chen et al., 2019; Du et al., 2020). Since Se_1-*x*_S_*x*_ cathode materials exhibit similar electrochemical behaviors to S, the strategies of immobilizing S should also be effective for Se_1-*x*_S_*x*_ (Luo et al., 2014). At present, the main host materials of Se_1-*x*_S_*x*_ are carbonaceous materials (Sun et al., 2021), such as hollow carbon spheres (Xu et al., 2015; Hu et al., 2020), mesoporous carbon (Han et al., 2019), carbon nanotubes (Fan et al., 2018; Guan et al., 2019; Shen et al., 2020), carbon fiber (Chen et al., 2014; Zhang et al., 2017), graphene (Tang et al., 2016; Chen et al., 2019) and carbonized polyacrylonitrile (Li et al., 2018). Generally, expect serving as hosts for Se_1-*x*_S_*x*_, these carbonaceous materials play another dual role of establishing conductive frameworks to facilitate ions/electrons transport and inhibiting the shuttle effect (Hu et al., 2020). Nevertheless, the physical adsorption ability of nonpolar pristine carbon materials to polar intermediates is too weak to effectively prevent the dissolution and diffusion of intermediate (Sun et al., 2016; Nazarian-Samani et al., 2021). Research shows heteroatom-doped (B, N, O, *etc*.) carbon hosts can effectively improve the electrochemical performance of Li-Se_1-*x*_S_*x*_ batteries due to the strong chemical affinity of polarized carbon surface, which can significantly trap the soluble intermediates to inhibit the shuttle effect and side reactions in the electrolyte (Guo et al., 2016; Zhang et al., 2017; Fan et al., 2018; He et al., 2018; Sun et al., 2021).

**FIGURE 1:**
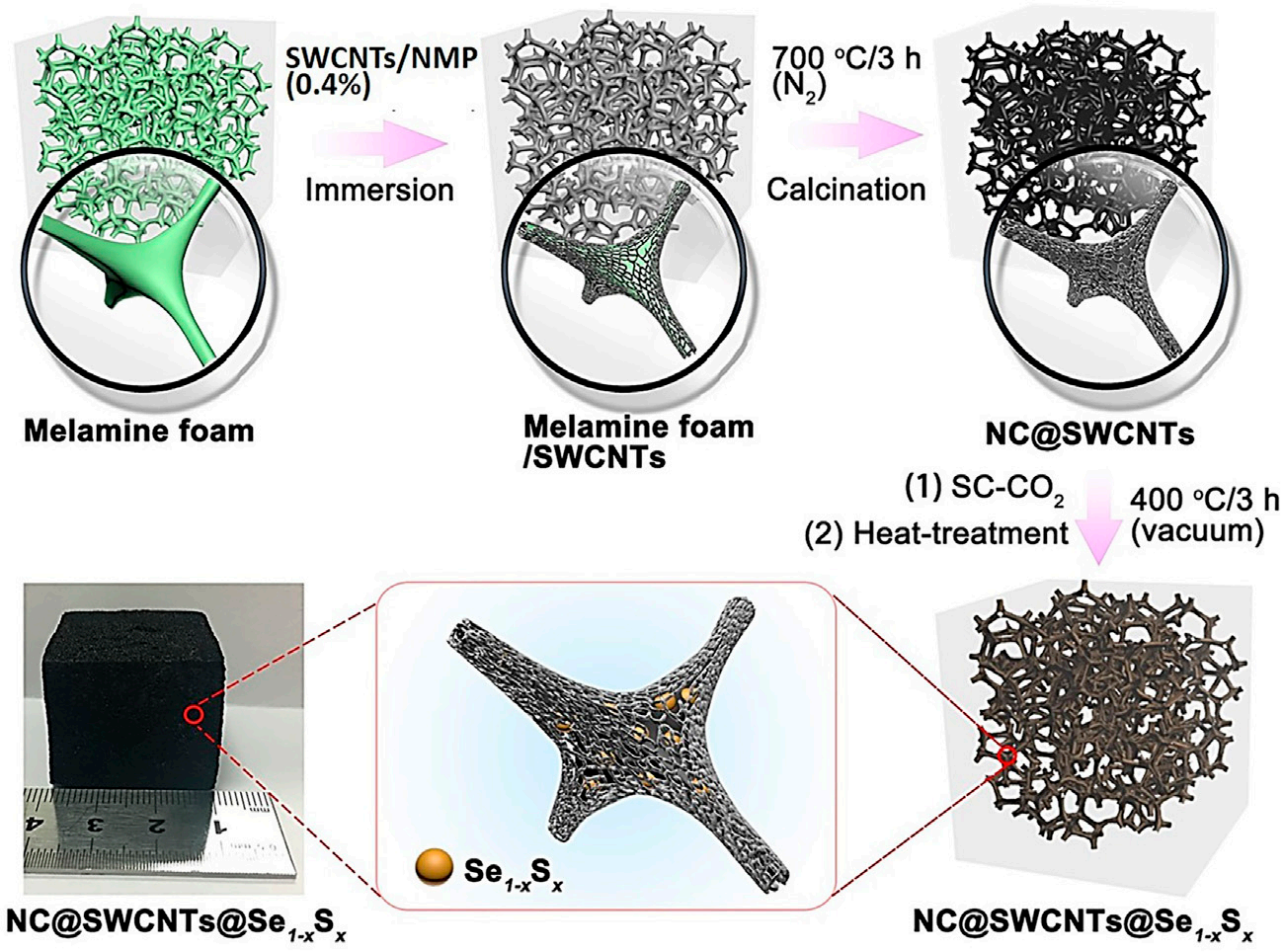
Schematic illustration of the synthetic process of NC@SWCNTs@Se_1-*x*_S_*x*_.

Herein, a series of Se_1-*x*_S_*x*_ cathode materials with optimized Se/S ratio are incorporated into N-doped three-dimensional (3D) porous carbon matrix to form novel freestanding Se_1-*x*_S_*x*_ foamy cathodes (NC@SWCNTs@Se_1-*x*_S_*x*_) with the assistance of supercritical CO_2_ fluid (Figure 1). In carbonate-based electrolyte, NC@SWCNTs@Se_1-*x*_S_*x*_ cathodes exhibit single-phase transformation during charge/discharge. Benefiting from the rationally designed structure and chemical composition, NC@SWCNTs@Se_1-*x*_S_*x*_ cathodes with high conductivity and strong adsorption present superior electrochemical performance.

## Experimental Section

### Preparation of NC@SWCNTs

Melamine foam (3 cm × 3 cm × 3 cm) was washed with anhydrous ethanol and dried in an oven at 80°C for 12 h, then immersed in SWCNTs/NMP suspension (0.4%). After 6 h, the melamine foam impregnated with SWCNTs/NMP suspension was taken out and dried in a vacuum oven at 80°C for 24 h to obtain the precursor of melamine/SWCNTs. The above precursor was calcined at 700°C under flowing N_2_ atmosphere for 3 h to obtain NC@SWCNTs host.

### Preparation of NC@SWCNTs@Se_1-x_S_x_

NC@SWCNTs@Se_1-*x*_S_*x*_ composites were prepared with the help of supercritical CO_2_ (SC-CO_2_) fluid, which is reported in our previous works (Fang et al., 2018a; Fang et al., 2018b; Fang et al., 2020). Firstly, S and Se powders with different molar ratios (S:Se = 7:3, 8:2, 9:1) were put into stainless-steel milling jars, respectively. The pre-mixed Se and S mixture was obtained after ball milling (500 rpm) for 12 h. Subsequently, 0.6 g pre-mixed Se and S mixture and a piece of NC@SWCNTs (3 cm × 3 cm × 3 cm, ∼0.4 g) were put into a stainless-steel jar. Then, CO_2_ was pumped into the jar until the gaseous pressure reached 8.5 MPa. After the jar was kept at 32°C for 24 h, NC@SWCNTs@Se_1-*x*_S_*x*_ precursor was obtained by rapidly releasing CO_2_. Then, NC@SWCNTs@Se_1-*x*_S_*x*_ precursor was sealed in a quartz glass tube under vacuum. Finally, the sealed quartz glass tube was heated to 400°C for 3 h to obtain NC@SWCNTs@Se_1-*x*_S_*x*_. The samples with different Se/S molar ratios were labeled as NC@SWCNTs@Se_0.3_S_0.7_, NC@SWCNTs@Se_0.2_S_0.8_ and NC@SWCNTs@Se_0.1_S_0.9_, respectively. For comparison, NC@SWCNTs hosts impregnated with Se or S were prepared by using the same SC-CO_2_ method and named NC@SWCNTs@Se and NC@SWCNTs@S, respectively.

### Materials Characterizations

The morphologies and microstructures of samples were observed on field-emission scanning electron microscopy (FE-SEM, Hitachi S-4800) and transmission electron microscopy (TEM, FEI Tecnai G2 F30) equipped with an energy-dispersive spectroscopy (EDS) detector. X-ray diffraction (XRD) patterns were recorded on Rigaku Ultima IV powder X-ray diffractometer by using Cu Kα radiation (*λ* = 0.15418 nm). Raman spectra were performed by Renishaw In*Via* Raman spectrometer (*λ* = 532 nm). Thermogravimetric analysis (TGA) was conducted on SDT Q600 analyzer (TA Instruments) under a flowing Ar atmosphere.

### Electrochemical Measurements

NC@SWCNTs@Se_1-*x*_S_*x*_ cathodes were cut into disks of 15 mm in diameter and 2 mm in height. CR2025 coin-type cells were assembled in an Ar-filed glove box (MIKROUNA, moisture <1.0 ppm, oxygen <1.0 ppm) with NC@SWCNTs@Se_1-*x*_S_*x*_ composites as cathodes, commercial microporous polypropylene membrane (Celgard 2400) as separator, and lithium metal as anode. A solution of 1.0 M LiPF_6_ in a co-solvent of ethylene carbonate (EC) and dimethyl carbonate (DMC) (1:1, volume ratio) was used as electrolyte. The dosage of electrolyte in coin-type cells is 15 μl mg^−1^ (based on the mass of Se_1-*x*_S_*x*_). Li-Se_1-*x*_S_*x*_ cells were cycled in the voltage range of 1.0–3.0 V on a battery testing system (Shenzhen Neware Technology Co. Ltd.). Cyclic voltammetry (CV) was performed on a CHI650B electrochemical workstation (Chenhua, Shanghai, China).

## Results and Discussion

**FIGURE 2:**
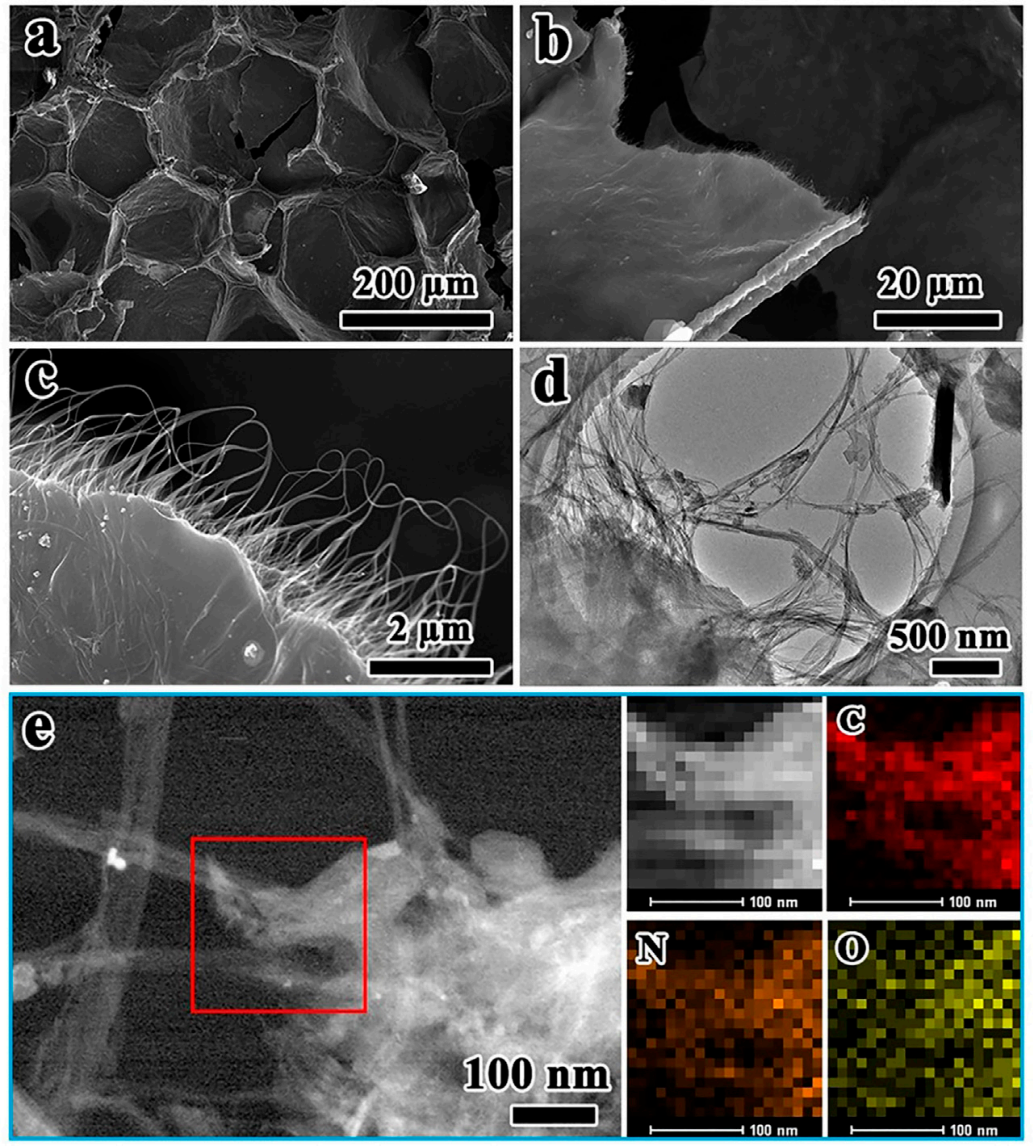
**(A–C)** SEM images and **(D)** TEM image of NC@SWCNTs. **(E)** STEM image of NC@SWCNTs and the corresponding mapping images.

The morphology and microstructure of NC@SWCNTs host are characterized by SEM and TEM as illustrated in Figure 2. As vividly depicted in Figure 2A and Supplementary Figure S1, NC@SWCNTs host exhibits a 3D honeycombed network structure, fully inheriting the 3D interconnected framework of melamine foam. Local magnification SEM images (Figures 2B,C) demonstrate that numerous interlaced SWCNTs are covered the surface of melamine foam derived carbon skeletons, as well as SWCNTs are formed into small sheets between carbon skeletons. This unique interconnecting structure not only endows NC@SWCNTs a highly conductive 3D network to accelerate the electron/ion transport, but also effectively enhances the mechanical strength and flexibility of NC@SWCNTs host. Moreover, TEM results (Figure 2D) further indicate that SWCNTs are crisscrossed in carbon skeletons, forming an intertwined 3D network structure. On the basis of EDS results (Figure 2E), the main elements in NC@SWCNTs are C, O and N, which are uniformly distributed in NC@SWCNTs. Notably, N signal is derived from melamine foam since melamine has high content of N. According to the above analysis, NC@SWCNTs host has a typical 3D network structure that is composed of SWCNTs-coated N-doped carbon skeleton derived from melamine foam and wafery sheets interwoven by SWCNTs. The pores and layer gaps in NC@SWCNTs host are conducive to loading more Se_1-*x*_S_*x*_ active materials. Meanwhile, the 3D interconnected conductive network framework can not only effectively promote redox kinetics, but also endow NC@SWCNTs host with strong mechanical properties to buffer the volume expansion during cycling. Additionally, the doped N is also beneficial to the adsorption of intermediates.

After Se_1-*x*_S_*x*_ impregnation, compared to NC@SWCNTs host, NC@SWCNTs@Se_1-*x*_S_*x*_ composites well maintain the original morphology of NC@SWCNTs (Supplementary Figure S2). Moreover, no discernible Se_1-*x*_S_*x*_ particles can be found at the surface of NC@SWCNTs. Additionally, according to EDS mapping results, the C, N, Se and Se signals are overlapped well, suggesting Se_1-*x*_S_*x*_ composites are uniformly permeated into the pores and layer gaps of NC@SWCNTs host with the assistance of SC-CO_2_ due to the good permeability, excellent diffusivity and high solubility of SC-CO_2_. Furthermore, elemental analyses (Supplementary Table S1) of NC@SWCNTs@Se_1-*x*_S_*x*_ show that molar ratios of Se to S in NC@SWCNTs@Se_1-*x*_S_*x*_ conform to the design values.

**FIGURE 3:**
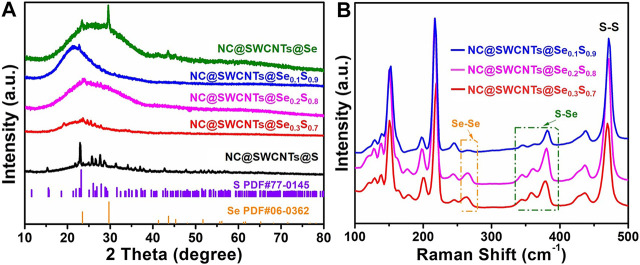
**(A)** XRD patterns of NC@SWCNTs@S, NC@SWCNTs@Se_0.3_S_0.7_, NC@SWCNTs@Se_0.2_S_0.8_, NC@SWCNTs@Se_0.1_S_0.9_ and NC@SWCNTs@Se. **(B)** Raman spectra of NC@SWCNTs@Se_0.3_S_0.7_, NC@SWCNTs@Se_0,2_S_0.8_ and NC@SWCNTs@Se_0.1_S_0.9_.

NC@SWCNTs@Se_1-*x*_S_*x*_ composites are further revealed by XRD and Raman analysis. As illustrated in Figure 3A, all the samples have a wide peak in 2θ ranging from 15 to 40^o^, corresponding to the existence of NC@SWCNTs. Meanwhile, the characteristic diffraction peaks of Se and S are clearly observed in NC@SWCNTs@Se and NC@SWCNTs@S samples, respectively. With the introduction of Se, no characteristic diffraction peak of Se is detected in NC@SWCNTs@Se_1-*x*_S_*x*_ composites. However, some characteristic diffraction peaks of S with low intensity can be still observed, indicating a small amount of Se may occupy S position and further form Se_1-*x*_S_*x*_ in NC@SWCNTs host (Yao et al., 2017). To further investigate the bond between Se and S, Raman spectra were depicted in Figure 3B. Apparently, all the samples have three characteristic peaks located at 260, 375 and 470 cm^−1^, respectively, which are assigned to Se-Se, Se-S and S-S stretching vibrations, (Chen et al., 2019; Pham et al., 2019). With increasing Se content in Se_1-*x*_S_*x*_, the strength of Se-Se and Se-S bonds are simultaneously increased, whereas the strength of S-S bonds are gradually decreased. Thus, it could be concluded that Se_1-*x*_S_*x*_ composites are successfully synthesized.

To further inspect the Se_1-*x*_S_*x*_ content and thermal stability of NC@SWCNTs@Se_1-*x*_S_*x*_ composites, TGA tests are performed as shown in Supplementary Figure S3. According to TG results, NC@SWCNTs@S and NC@SWCNTs@Se exhibit the lowest and highest onset decomposition temperatures of ∼130 and ∼300°C, respectively. Meanwhile, the onset decomposition temperatures of NC@SWCNTs@Se_0.3_S_0.7_, NC@SWCNTs@Se_0.2_S_0.8_, and NC@SWCNTs@Se_0.1_S_0.9_ are between NC@SWCNTs@S and NC@SWCNTs@Se samples, and gradually increase with increasing Se content in Se_1-*x*_S_*x*_. It is because the thermal stability of Se is higher than that of S, the higher the content of Se in Se_1-*x*_S_*x*_, the higher the thermal stability of solid solution. Moreover, the distinct weight losses exist in all the samples, corresponding to the active material in samples. Therefore, the actual contents of S, Se_0.3_S_0.7_, Se_0.2_S_0.8_, Se_0.1_S_0.9_, and Se in NC@SWCNTs@S, NC@SWCNTs@Se_0.3_S_0.7_, NC@SWCNTs@Se_0.2_S_0.8_, NC@SWCNTs@Se_0.1_S_0.9_, and NC@SWCNTs@Se are 54.8, 58.7, 52.5, 58.4, and 56.8%, respectively, which are close to the design value of ∼60%.

**FIGURE 4:**
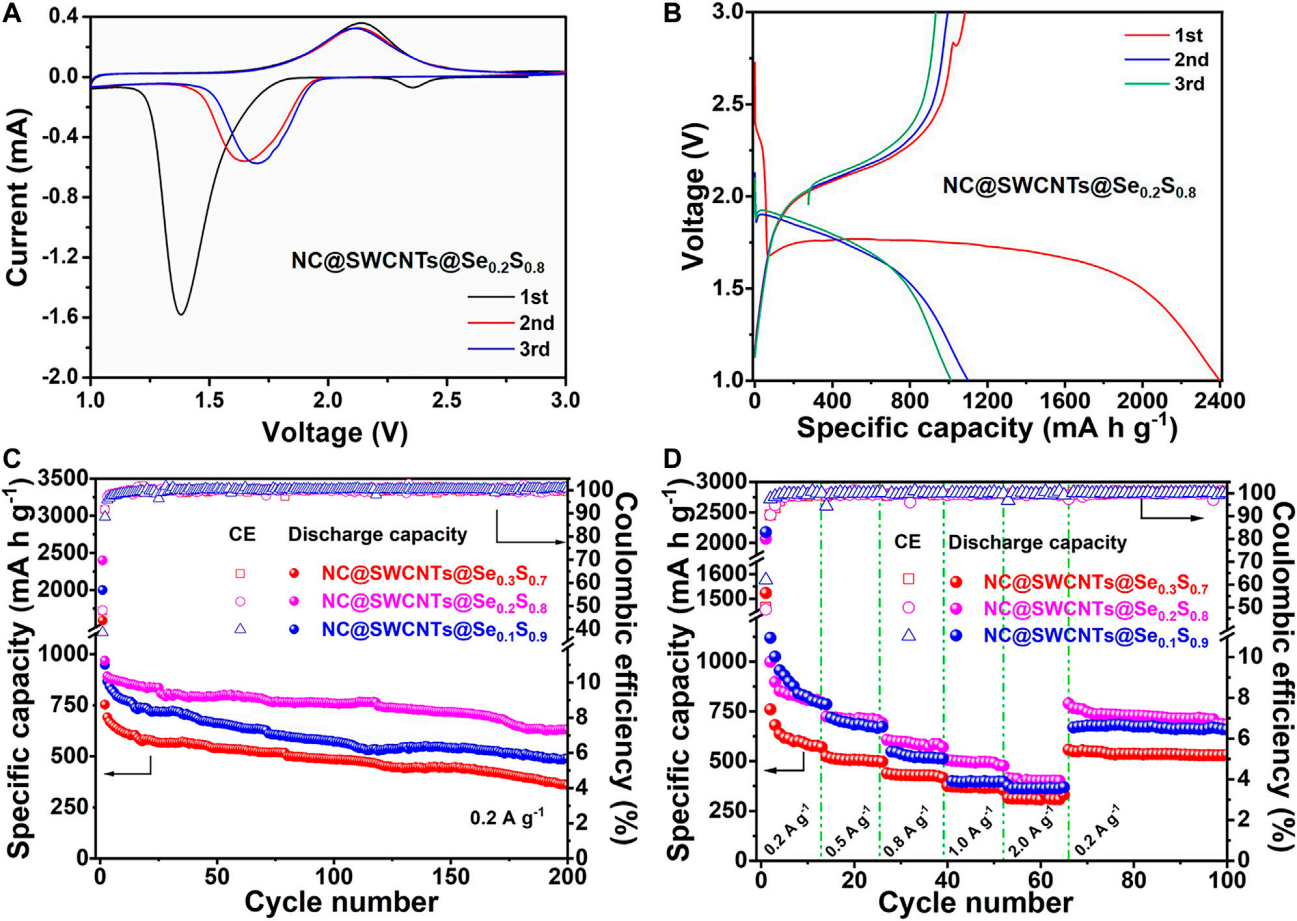
**(A)** CV curves of the NC@SWCNTs@Se_0.2_S_0.8_ cathode. **(B)** Charge/discharge curves of the NC@SWCNTs@Se_0.2_S_0.8_ cathode at 0.2 A g^−1^. **(C)** Cycle performances and **(D)** rate performances of NC@SWCNTs@Se_1-*x*_S_*x*_ cathodes.

In order to evaluate the electrochemical performance of NC@SWCNTs@Se_1-*x*_S_*x*_ composites, NC@SWCNTs@Se_1-*x*_S_*x*_ composites are employed as freestanding cathodes in Li-Se_1-*x*_S_*x*_ batteries with carbonate-based electrolyte (LiPF_6_-EC/DMC). Figure 4A and Supplementary Figure S4 show initial three cyclic voltammetry (CV) curves of NC@SWCNTs@Se_1-*x*_S_*x*_ cathodes at a scanning rate of 0.1 mV s^−1^ in the potential window from 1.0 to 3.0 V versus Li/Li^+^. At the initial scan, a sharp reduction peak at ∼1.38 V, a small reduction peak at ∼2.37 V, and a broadened oxidation peak at ∼2.14 V are clearly observed. The small reduction peak at ∼2.37 V disappears after the first scan, while the sharp reduction peak at ∼1.38 V shifts to ∼1.7 V during the subsequent scan. The peak shift indicates the activation process during the first lithification process, and the polarization is effectively reduced thereafter (Luo et al., 2014; Zhu et al., 2018). The subsequent CV curves are well overlapped after the first scan, indicating the good cyclability and reversibility of NC@SWCNTs@Se_0.2_S_0.8_ cathode (Guo et al., 2019). It should be mentioned that the CV curves of NC@SWCNTs@Se_1-*x*_S_*x*_ cathodes are obviously different from S cathode, indicating the introduction of Se changes the electrochemical reaction process of S that is conducive to its stable work in carbonate-based electrolytes. Moreover, galvanostatic charge-discharge curves (Figure 4B and Supplementary Figure S5) of NC@SWCNTs@Se_1-*x*_S_*x*_ cathodes are consistent with CV results. During the first discharge process, there are two plateaus: one is an extremely short plateau at ∼2.38 V, and another is a long plateau at ∼1.75 V. In the subsequent cycles, the short plateau at ∼2.38 V disappears, while the long plateau at ∼1.75 V becomes a little steeper and shifts to ∼1.88 V. The short plateau at ∼2.38 V is attributed to the transformation of Se_0.2_S_0.8_ to polysulfides/polyselenides intermediates. And the disappearance of the short plateau is probably due to the dissolution of intermediates into the electrolyte (Li et al., 2015). Meanwhile, the long plateau at 1.75–1.88 V is assigned to the conversion of polysulfides/polyselenides to Li_2_S/Li_2_Se (Luo et al., 2014). During the charge process, there is only one sloping plateau at ∼2.12 V, corresponding to the conversion of Li_2_Se/Li_2_S to Se_0.2_S_0.8_.

Figure 4C shows the cyclic performance of NC@SWCNTs@Se_1-*x*_S_*x*_ cathodes with different Se/S ratios at a current density of 0.2 A g^−1^. NC@SWCNTs@Se_0.2_S_0.8_ cathode delivers the highest initial discharge capacity (2,398.5 mA h g^−1^) among NC@SWCNTs@Se_0.3_S_0.7_, NC@SWCNTs@Se_0.2_S_0.8_ and NC@SWCNTs@Se_0.1_S_0.9_ samples. The initial discharge capacity exceeds the theoretical capacity may be attributed to side reactions and the formation of SEI layer on the surface of electrode (Luo et al., 2014). After 200 cycles, the reversible capacities of NC@SWCNTs@Se_0.3_S_0.7_, NC@SWCNTs@Se_0.2_S_0.8_ and NC@SWCNTs@Se_0.1_S_0.9_ samples are 490, 632 and 360 mA h g^−1^ with the corresponding capacity retentions of 51.7, 65.3 and 47.9%, respectively. Obviously, NC@SWCNTs@Se_0.2_S_0.8_ cathode exhibits the superior cyclic stability. In addition, the rate capabilities of NC@SWCNTs@Se_1-*x*_S_*x*_ cathodes at different current densities are presented in Figure 4D. Compare to other samples, NC@SWCNTs@Se_0.2_S_0.8_ cathode demonstrates the best rate performance. The reversible rate capacities of NC@SWCNTs@Se_0.2_S_0.8_ cathode are 998.4, 723.7, 606.8, 506.1, and 415.0 mA h g^−1^ at the current density of 0.2, 0.5, 0.8, 1.0 and 2.0 A g^−1^, respectively. When the current density switches back to 0.5 A g^−1^, the reversible discharge capacity of NC@SWCNTs@Se_0.2_S_0.8_ cathode reverts to the initial value. Moreover, as shown in Supplementary Table S2 and Supplementary Figure S4, NC@SWCNTs@Se_0.2_S_0.8_ cathode with Se loading of as high as 4.4 mg cm^−2^ (a relevant areal capacity of as high as 2.78 mA h cm^−2^) can surpass most reported Se_1-*x*_S_*x*_ cathodes (Luo et al., 2014; Li et al., 2015; Guo et al., 2016; Wei et al., 2016; Li et al., 2017; Yao et al., 2017; Zhang et al., 2017; Hu et al., 2018; Li et al., 2018; Zhu et al., 2018). Such remarkable electrochemical performance of NC@SWCNTs@Se_0.2_S_0.8_ cathode mainly is due to the following reasons: 1) Se and S in Se_0.2_S_0.8_ solid solution play different roles: Se can significantly improve the electrical conductivity, while S can greatly enhance capacity. 2) N-doped 3D porous carbon matrix and interlaced SWCNTs not only provide storage space for Se_1-*x*_S_*x*_, but also effectively reinforce the structural stability, and further promote the cycling stability of NC@SWCNTs@Se_1-*x*_S_*x*_ cathodes.

## Conclusion

In summary, a series of rationally designed freestanding NC@SWCNTs@Se_1-*x*_S_*x*_ cathodes with 3D interconnected porous structure are developed with the assistance of supercritical CO_2_ fluid. NC@SWCNTs host with 3D network structure serves as an effective matrix for encapsulating Se_1-*x*_S_*x*_ as well as facilitating ion/electron transport and redox kinetics. Benefiting from the rationally designed structure and optimized chemical composition, NC@SWCNTs@Se_0.2_S_0.8_ cathode exhibits excellent cycling stability (632 mA h g^−1^ at 0.2 A g^−1^ at 200 cycle) and remarkable rate performance (415 mA h g^−1^ at 2 A g^−1^) in carbonate-based electrolyte. This work offers a feasible approach to develop high-performance Se_1-*x*_S_*x*_ cathodes for advanced Li-Se_1-*x*_S_*x*_ batteries.

## Data Availability

The original contributions presented in the study are included in the article/Supplementary Material, further inquiries can be directed to the corresponding author.
