# Supplementary material for: Supercritical CO2 Synthesis of Freestanding Se1-xSx Foamy Cathodes for High-Performance Li-Se1-xSx Battery
Source: Front Chem. 2021 Jul 28;9:738977. doi: 10.3389/fchem.2021.738977 (PMC8355597; doi:10.3389/fchem.2021.738977)
Supplement: Supplementary file 1 [file DataSheet1.docx]

Supplementary Material

Supercritical CO_2_ Synthesis of Freestanding Se_1-_*_x_*S*_x_* Foamy Cathodes for High-Performance Li-Se_1-_*_x_*S*_x_* Battery

Chengwei Lu^1^, Ruyi Fang^1^, Kun Wang^1^, Zhen Xiao^2^, G.Gnana Kumar^3^, Yongping Gan ^1^, Xinping He^1^, Hui Huang^1^, Wenkui Zhang^1^, Yang Xia^1*^

^1^ College of Materials Science and Engineering, Zhejiang University of Technology, Hangzhou 310014, China

^2^ Institute of Optoelectronic Materials and Devices, China Jiliang University, Hangzhou, 310018, China

^3^ Department of Physical Chemistry, School of Chemistry, Madurai Kamaraj University, Madurai 625021, India

*** Correspondence:**Yang Xia
nanoshine@zjut.edu.cn


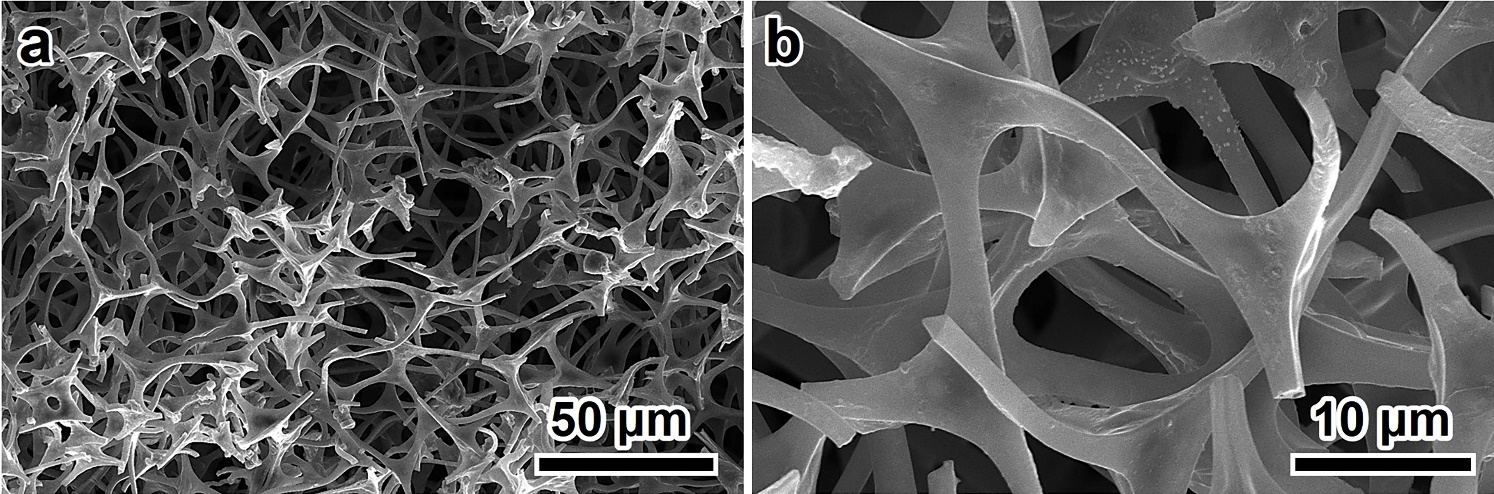


**Figure S1.** SEM images of melamine foam-derived N-doped carbon (NC).


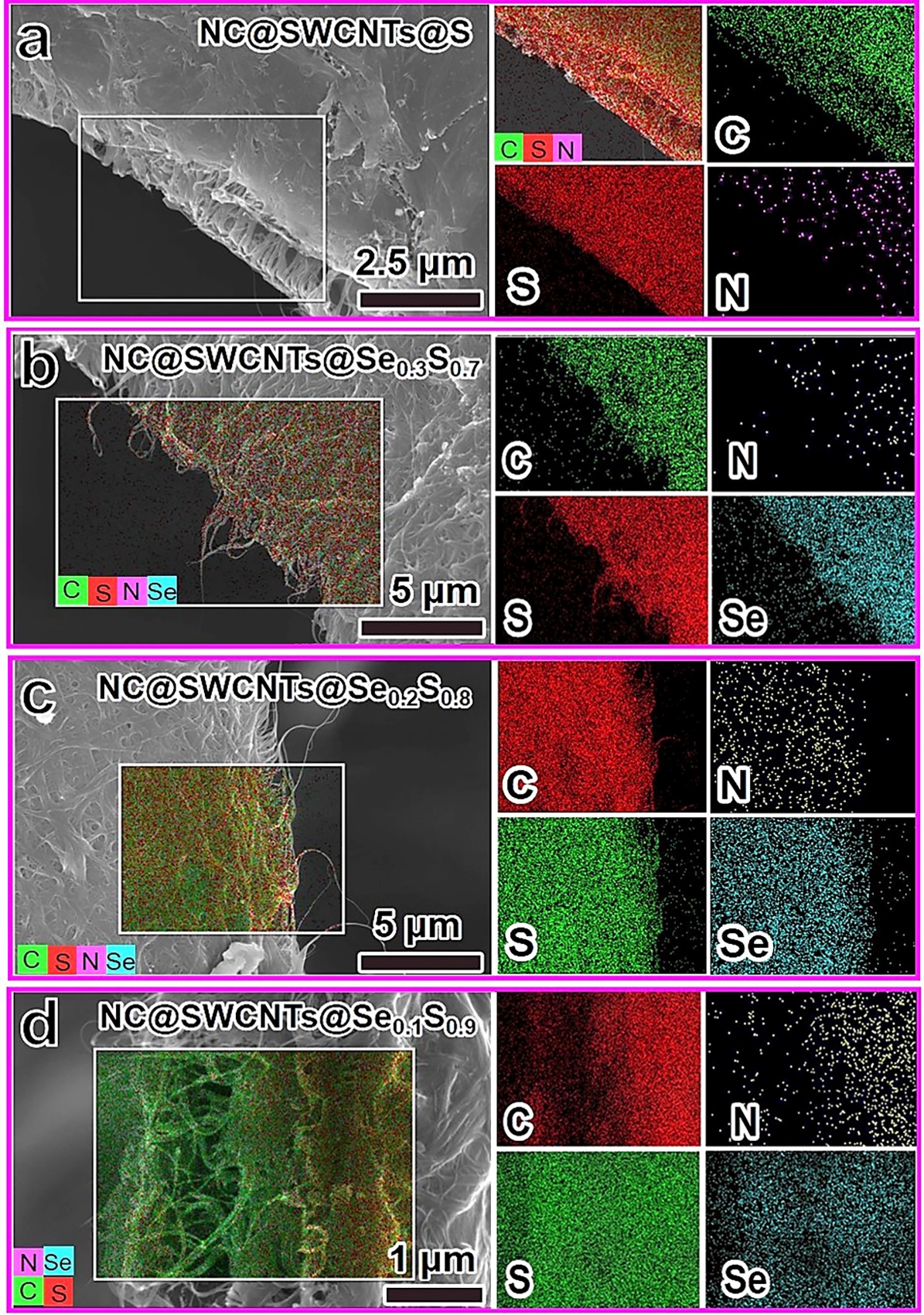


**Figure S2.** SEM images of NC@SWCNTs@Se_1-_*_x_*S*_x_* composites and the corresponding mapping images: (a) NC@SWCNTs@S, (b) NC@SWCNTs@Se_0.3_S_0.7_. (c) NC@SWCNTs@Se_0.2_S_0.8_. (d) NC@SWCNTs@Se_0.1_S_0.9_.

**Table S1.** Elemental analyses of NC@SWCNTs@Se_1-_*_x_*S*_x_*.

| **Sample** | **Atomic fraction (%)** | | | | |
| --- | --- | --- | --- | --- | --- |
|  | **C** | **N** | **O** | **S** | **Se** |
| NC@SWCNTs@Se_0.1_S_0.9_ | 44.2 | 14.8 | 7.4 | 30.1 | 3.5 |
| NC@SWCNTs@Se_0.2_S_0.8_ | 45.4 | 15.1 | 5.5 | 27.1 | 6.9 |
| NC@SWCNTs@Se_0.3_S_0.7_ | 46.0 | 16.0 | 8.2 | 21.0 | 8.8 |


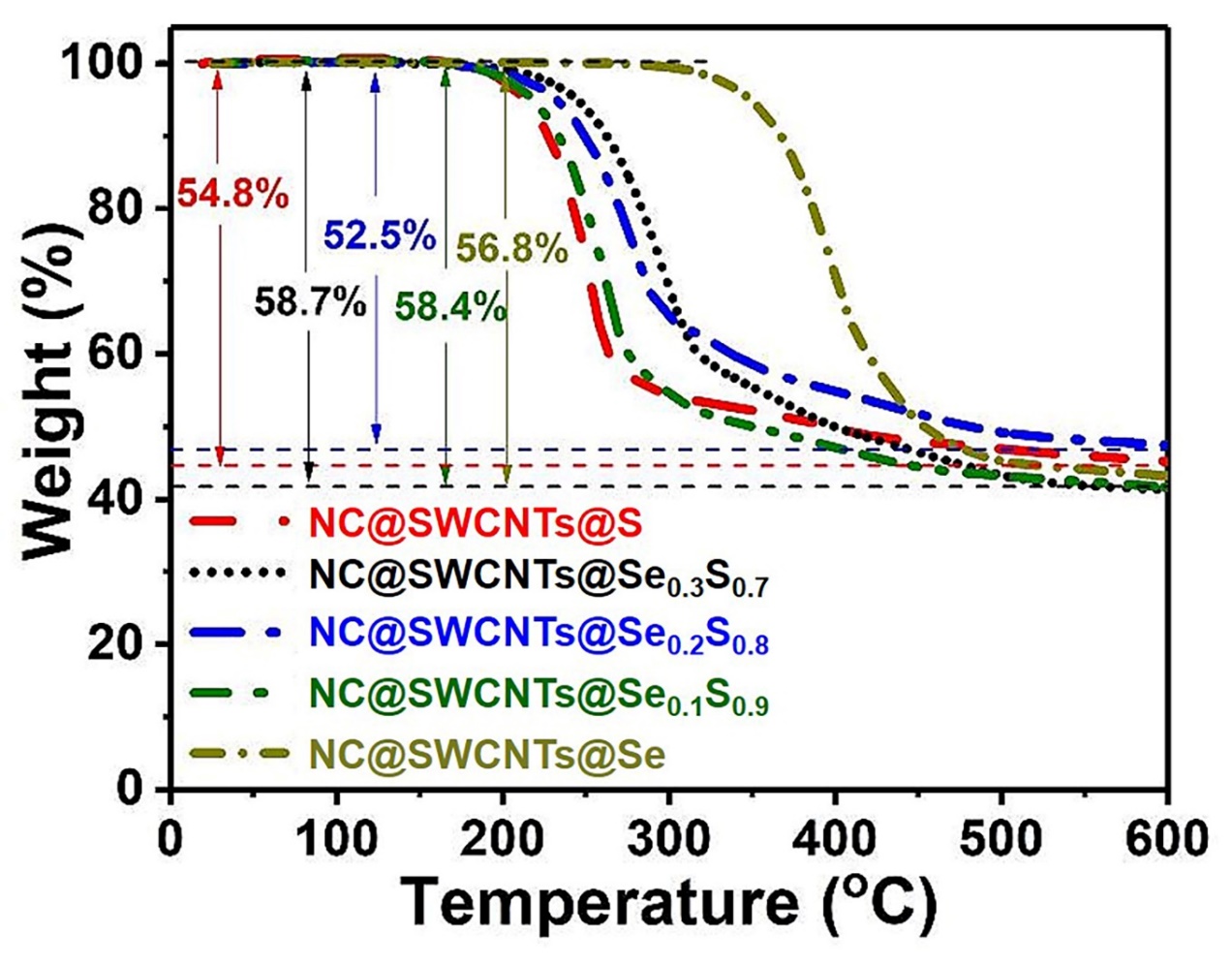


**Figure S3.** TGA curves of NC@SWCNTs@S, NC@SWCNTs@Se and NC@SWCNTs@Se_1-_*_x_*S*_x_*.


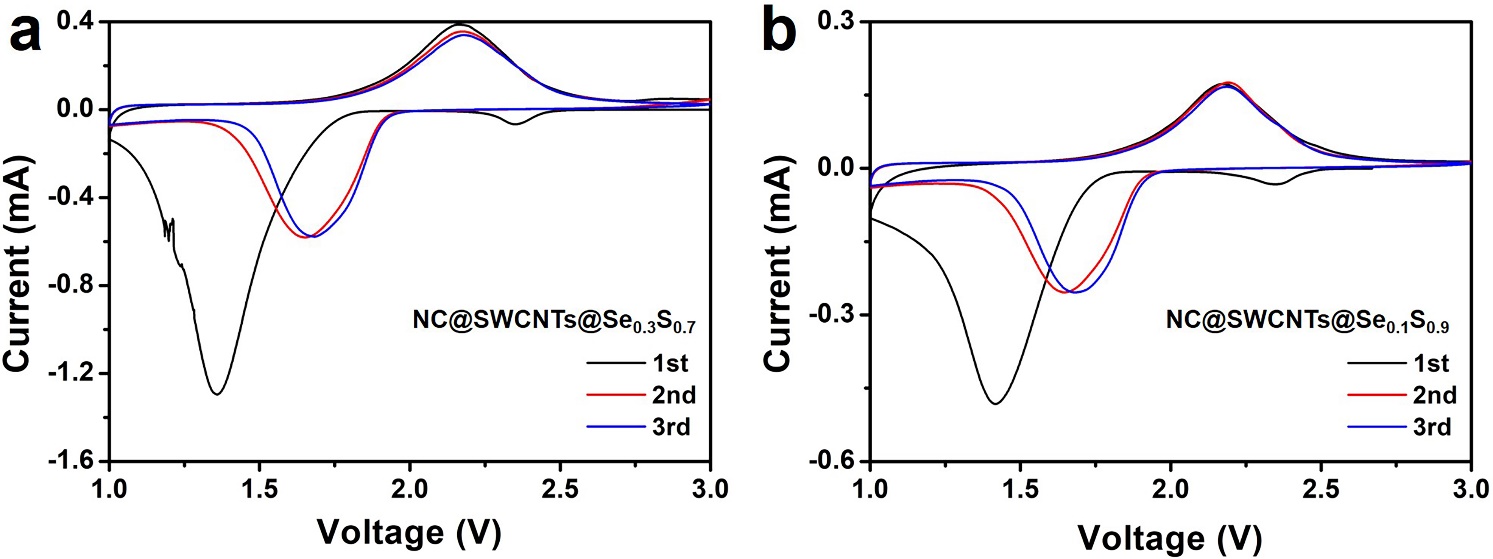


**Figure S4.** CV curves of (a) the NC@SWCNTs@Se_0.3_S_0.7_ cathode and (b) NC@SWCNTs@Se_0.1_S_0.9_ cathode.


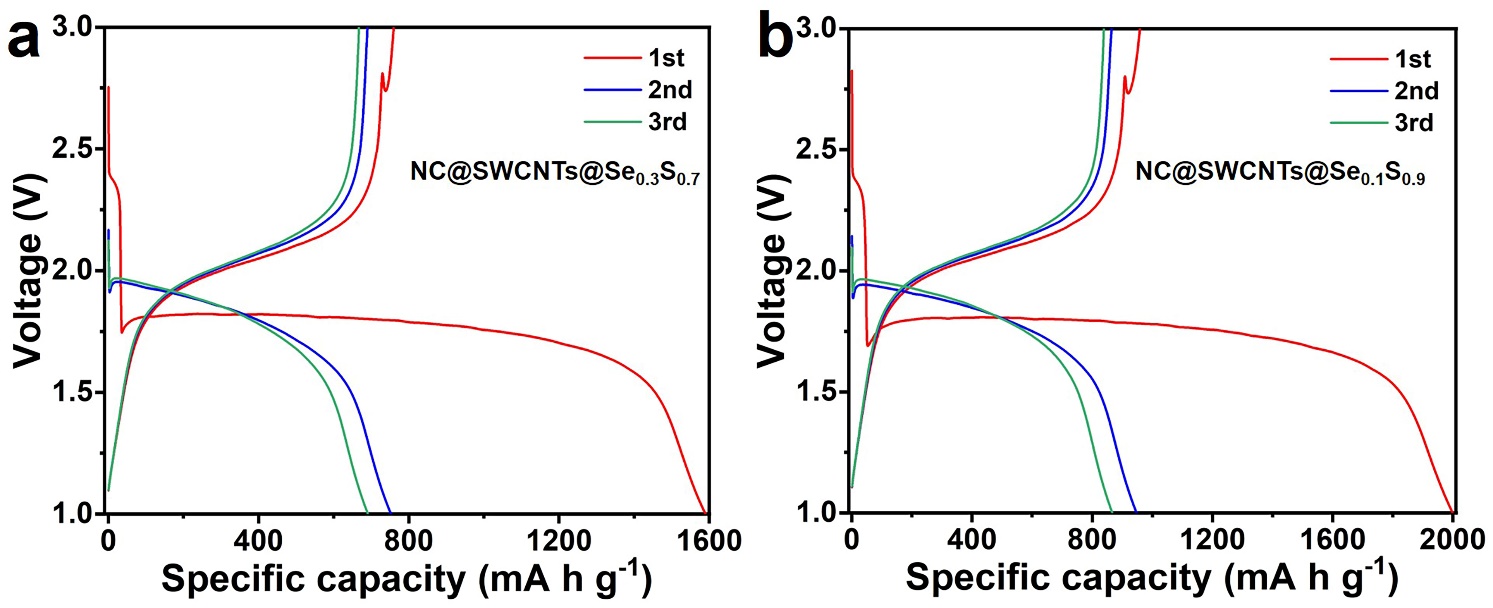


**Figure S5.** Charge/discharge curves of (a) the NC@SWCNTs@Se_0.3_S_0.7_ cathode and (b) the NC@SWCNTs@Se_0.1_S_0.9_ cathode at 0.2 A g^-1^.

**Table S2.** Comparison between NC@SWCNTs@Se_0.2_S_0.8_ cathode and state-of-the-art Se_1-_*_x_*S*_x_* cathodes from the published literatures (Luo et al., 2014; Li et al., 2015; Guo et al., 2016; Wei et al., 2016; Li et al., 2017; Yao et al., 2017; Zhang et al., 2017; Hu et al., 2018; Li et al., 2018; Zhu et al., 2018).

| **Sample** | **Mass**  **Loading**  **(mg cm^-2^)** | **Current**  **density** | **Initial**  **capacity**  **(mA h g^-1^)** | **Reversible**  **capacity**  **(mA h g^-1^**  **@cycles)** | **Areal**  **capacity**  **(mA h cm^-2^)** |
| --- | --- | --- | --- | --- | --- |
| S_0.87_Se_0.13_/CPAN | 1.14 | 0.3 A g^-1^ |  | 989@200 | 1.13 |
| S_0.6_Se_0.4_@CNFs | 0.9 | 0.1 A g^-1^ | 892 | 450@100 | 0.40 |
| S_0.94_Se_0.06_/C | 0.8-1.5 | 0.2 A g^-1^ | 1755 | 1090@200 | 1.25 |
| pPAN/SeS_2_ | ~2 | 0.5 A g^-1^ | 1451 | 1020@100 | 2.04 |
| SeS_0.1_/NCPAN | 3 | 0.05 C | 1387 | 276@100 | 0.83 |
| SeS_0.7_/CPAN | 1.2 | 0.6 A g^-1^ |  | 780@1200 | 0.94 |
| CMK-3/SeS_2_@PDA | 2.6-3 | 0.2 A g^-1^ | 1234 | 783@150 | 2.20 |
| Se_2_S_5_/MCM | 0.5-0.8 | 0.5 C | 1150.6 | 796.4@100 | 0.52 |
| CoS_2_@LRC/SeS_2_ | 2.3-2.5 | 0.2 A g^-1^ | 1015 | 745@100 | 1.79 |
| SeS_2_@ISMC | 1 | 0.5 A g^-1^ | 858 | 486@200 | 0.49 |
| **NC@SWCNTs@Se_0.2_S_0.8_**  **(This work)** | **4.4** | **0.2 A g^-1^** | **2398.5** | **632@200** | **2.78** |


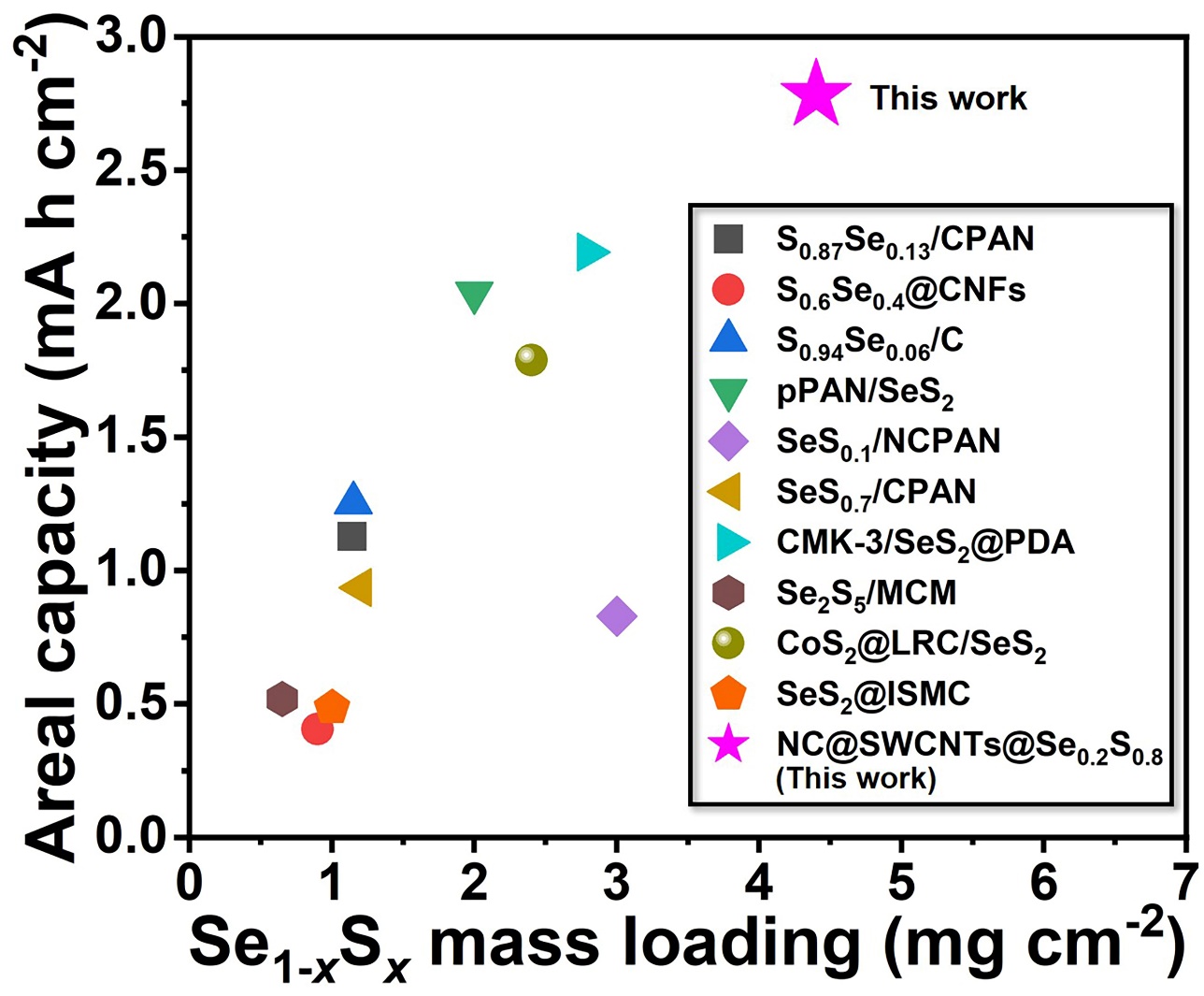


**Figure S6.** Comparison between NC@SWCNTs@Se_0.2_S_0.8_ cathode and state-of-the-art Se_1-_*_x_*S*_x_* cathodes from the published literature (Luo et al., 2014; Li et al., 2015; Guo et al., 2016; Wei et al., 2016; Li et al., 2017; Yao et al., 2017; Zhang et al., 2017; Hu et al., 2018; Li et al., 2018; Zhu et al., 2018).

# References

Guo, S.-P., Li, C.-X., Chi, Y., Ma, Z., and Xue, H.-G. (2016). Novel 3-D network SeS*_x_*/NCPAN composites prepared by one-pot in-situ solid-state method and its electrochemical performance as cathode material for lithium-ion battery. *J. Alloy. Compd.* 664, 92-98. doi: 10.1016/j.jallcom.2015.12.208.

Hu, J., Zhong, H., Yan, X., and Zhang, L. (2018). Confining selenium disulfide in 3D sulfur-doped mesoporous carbon for rechargeable lithium batteries. *Appl. Surf. Sci.* 457, 705-711. doi: 10.1016/j.apsusc.2018.06.296.

Li, X., Liang, J., Zhang, K., Hou, Z., Zhang, W., Zhu, Y., et al. (2015). Amorphous S-rich S_1−_*_x_*Se*_x_*/C (*x* ≤ 0.1) composites promise better lithium-sulfur batteries in a carbonate-based electrolyte. *Energ. Environ. Sci.* 8, 3181-3186. doi: 10.1039/C5EE01470K.

Li, Z., Zhang, J., Lu, Y., and Lou, X.W. (2018). A pyrolyzed polyacrylonitrile/selenium disulfide composite cathode with remarkable lithium and sodium storage performances. *Sci. Adv.* 4, eaat1687. doi: 10.1126/sciadv.aat1687.

Li, Z., Zhang, J., Wu, H.B., and Lou, X.W. (2017). An improved Li-SeS_2_ battery with high energy density and long cycle life. *Adv. Energy Mater.* 7, 1700281. doi: 10.1002/aenm.201700281.

Luo, C., Zhu, Y., Wen, Y., Wang, J., and Wang, C. (2014). Carbonized polyacrylonitrile-stabilized SeS*_x_* cathodes for long cycle life and high power density lithium ion batteries. *Adv. Funct. Mater.* 24, 4082-4089. doi: 10.1002/adfm.201303909.

Wei, Y., Tao, Y., Kong, Z., Liu, L., Wang, J., Qiao, W., et al. (2016). Unique electrochemical behavior of heterocyclic selenium-sulfur cathode materials in ether-based electrolytes for rechargeable lithium batteries. *Energy Storage Mater.* 5, 171-179. doi: 10.1016/j.ensm.2016.07.005.

Yao, Y., Zeng, L., Hu, S., Jiang, Y., Yuan, B., and Yu, Y. (2017). Binding S_0.6_ Se_0.4_ in 1D carbon nanofiber with C-S bonding for high-performance flexible Li-S batteries and Na-S batteries. *Small* 13, 1603513. doi: 10.1002/smll.201603513.

Zhang, J., Li, Z., and Lou, X.W. (2017). A freestanding selenium disulfide cathode based on cobalt disulfide-decorated multichannel carbon fiberswith enhanced lithium storage performance. *Angew. Chem. Int. Edit.* 56, 14107-14112. doi: 10.1002/ange.201708105.

Zhu, T., Pang, Y., Wang, Y., Wang, C., and Xia, Y. (2018). S_0.87_Se_0.13_/CPAN composites as high capacity and stable cycling performance cathode for lithium sulfur battery. *Electrochim. Acta* 281, 789-795. doi: 10.1016/j.electacta.2018.06.026.
